# Data-Driven Infectious Disease Control: Qualitative Study of Professionals’ Attitudes, Barriers, and Needs

**DOI:** 10.2196/81036

**Published:** 2025-11-17

**Authors:** Babette van Deursen, Cornelia H M van Jaarsveld, Tessa van der Bruggen, Stijn Raven, Julie E M Swillens, Aura Timen

**Affiliations:** 1 Department of Primary and Community Care Research Institute for Medical Innovation Radboud University Medical Center Nijmegen The Netherlands; 2 Department of Infectious Disease Control Public Health Service region Utrecht Zeist The Netherlands; 3 IQ Health Science Department Radboud University Medical Center Nijmegen The Netherlands

**Keywords:** attitude of health personnel, communicable disease control, data-driven decision making, public health informatics, qualitative research

## Abstract

**Background:**

Data-driven strategies are increasingly integrated into infectious disease control (IDC), enabling professionals to act in a timely and proactive manner; however, their implementation requires alignment with professionals’ needs. Little is known about professionals’ views on data-driven IDC.

**Objective:**

This study aims to assess IDC professionals’ knowledge, attitudes, and perceptions toward working in a data-driven manner, as well as their needs, facilitators, and barriers.

**Methods:**

We conducted exploratory online focus group discussions (FGDs) with IDC professionals from the Public Health Services (PHSs) in the Netherlands. FGDs were organized by profession, followed by a final mixed-group session that included medical doctors, nurses, infection preventionists, epidemiologists, policy advisors, project leaders, and managers working in IDC. The topic guide was based on the Attitude-Social Influence-Efficacy model and the Consolidated Framework for Implementation Research, complemented with questions on current practices within the PHS related to data-driven work (DDW). Framework and thematic analyses were performed.

**Results:**

Between September 2024 and January 2025, nine FGDs were conducted with 36 IDC professionals. Five main themes emerged: (1) context of the work environment, (2) interpretation of DDW in the context of IDC, (3) added value of DDW, (4) views on team participation, and (5) perspectives on development and implementation. While participants mentioned that some data-driven strategies were already implemented within the PHS, they observed that data were not often translated into action. Attitudes toward DDW varied across participants, particularly regarding its definition, application in daily practice, the importance of data interpretation by professionals, results, implementation, and added value. Participation in DDW varied within teams and was influenced by role, interest, workload, time, knowledge, and willingness to change. Participants also identified various facilitators, barriers, and needs at the individual, process, and organizational levels, such as a national approach of data-driven IDC guided by a shared vision, defined role assignments, and clear protocols for data registration.

**Conclusions:**

In this study, IDC professionals generally expressed a positive attitude toward DDW but also identified several barriers and needs for future implementation. The limited translation of data into action was viewed as untapped potential. To support effective data-driven IDC, we recommend investing in a supportive work environment that promotes a clear, shared definition of DDW, including defined roles and responsibilities. By doing so, IDC professionals can shift from reactive to strategic, data-informed action and be better equipped future public health threats.

## Introduction

Timely and evidence-informed action is critical for responding to public health threats. The COVID-19 pandemic highlighted this need, resulting in a growing demand among public health professionals to work in a more data-driven manner [1,2]. In the early stages of the pandemic, reliable data on the virus, its transmission, clinical course, and effective control measures were scarce. Gradually, more information and data became available. Infectious disease data were increasingly used for surveillance, monitoring public health interventions, and informing decision- and policymaking. The growing availability of data, combined with the high pressure to respond in a timely and accurate manner, elevated the role of surveillance data in public health decision- and policymaking. Not only public health professionals, but also policymakers, infectious disease control (IDC) team managers, and governments use data for policymaking [3]. In the Netherlands, IDC professionals of the Public Health Service (PHS) are typically responsible for decisions on outbreak control measures during localized outbreaks. In contrast, during the COVID-19 pandemic, national-level decisions were informed by a national expert outbreak management team, with the Dutch government having the final authority on control measures [4]. Despite the increasing reliance on data, several limitations became evident. Surveillance data were mostly not available in real time, and their quality and completeness varied [5]. As the pandemic progressed, the volume of information created an overload in data, which further increased the workload of IDC professionals, while timely understanding of the virus and its transmission remained crucial. Nevertheless, the urgency to adopt a more structured and sustainable data-driven approach in IDC has only grown, highlighting the need for better preparedness for future public health threats, including pandemics.

To date, there is no shared definition of “data-driven infectious disease control” in the Netherlands. For the purpose of this study, we define data-driven work (DDW) within IDC as follows: data-driven IDC involves using data in a systematic way to support policy and decision making, recognizing that data alone are not sufficient. Context, expertise, and interpretation transform data into meaningful information—data for action—which eventually supports policy and decision making. Within IDC, data-driven approaches can comprise various processes, including outbreak management, surveillance, policy development, intervention evaluation, strategic feedback, and operational optimization. Although IDC data have always been essential for professionals, DDW has not been standard practice within the Dutch PHS. Across the 25 PHSs in the Netherlands, various professionals, such as medical doctors, nurses, infection preventionists, epidemiologists, policy advisors, and managers, are involved in IDC activities. A national study conducted before the COVID-19 pandemic revealed that no formal agreements existed regarding defined tasks, time investment, role distribution, and responsibilities related specifically to infectious disease surveillance at the PHS level [6]. Furthermore, there were notable differences among PHSs in how surveillance was conducted, ranging from simple visual inspection of data and reliance on professional intuition to basic statistical analyses. Importantly, this earlier study was conducted before epidemiologists were structurally integrated into IDC teams. Since 2023, every PHS has had the opportunity to appoint an epidemiologist, supported by dedicated funding from the Ministry of Health, Welfare and Sport. However, no formal evaluation of the current state of infectious disease surveillance at PHS level has been conducted since their employment.

Despite the potential of data-driven IDC, its application remains limited. At the same time, the complexity and volume of available data continue to grow, which raises the need for a comprehensive and accessible overview of IDC data even more. As the Dutch PHSs have expressed their ambition to work in a data-driven manner in IDC, more data-driven initiatives are slowly being integrated [7]. However, the transition is complex. Working in a data-driven way involves not only adopting new behaviors at the individual level but also implementing a new working approach for IDC teams, which requires clear guidance and a facilitating work environment for implementation. Some barriers are known regarding data-driven decision making or data-driven surveillance in IDC, such as legal challenges in data sharing, time investment, insufficient human resources, and complex data integration [2,8]. In the current literature, little is known about the views of IDC professionals on data-driven IDC. To successfully adopt this new approach, it is necessary to understand which factors play a role in the adoption and implementation to facilitate and engage IDC professionals in the implementation process.

This study aims to address this gap. We explore both the behavioral and implementation factors of data-driven IDC. The objective of this study is to assess IDC professionals’ knowledge, attitudes, and perception toward DDW, as well as their needs, facilitators, and barriers to implementing this new working approach in their daily practice. By exploring these factors, we can align the approach of data-driven IDC with the requirements of IDC professionals, thereby facilitating implementation.

## Methods

### Ethical Considerations

We submitted the study protocol to the Medical Ethics Committee of Radboudumc, which determined that the study did not fall under the Dutch Medical Research Involving Human Subjects Act (Wet medisch-wetenschappelijk onderzoek met mensen [WMO]) [9]. According to this Act, research involving human participants requires ethical approval only if participants are subjected to procedures or are required to follow rules of behavior (WMO, Article 1b). As participants in this study were not subjected to WMO-governed procedures or behaviors, the committee granted an exemption from formal ethical approval (Medical Ethics Committee file number 2024-17475). All participants provided informed consent before participation, all results were deidentified, and no compensation was provided.

### Study Design

This qualitative study consisted of online focus group discussions (FGDs) with IDC professionals of the Dutch PHS. The focus groups had an exploratory approach to assess the knowledge, attitudes, and perceptions of IDC professionals regarding working in a data-driven manner.

### Recruiting and Selection Procedure

The IDC teams of the 25 PHSs were contacted by email with an information letter about the study. We invited medical doctors, nurses, infection preventionists, epidemiologists, policy advisors, project leaders, and managers to participate in the online FGDs, as they were most likely be involved in (future) DDW strategies. We used purposive sampling to recruit a wide variation of professionals, considering their work experience and the PHS region in the Netherlands. The FGDs were conducted with homogeneous group based on profession, while the final FGD involved a heterogeneous group. As the FGDs were conducted online, we aimed to recruit a minimum of 4 and maximum of 6 participants for each homogeneous FGD and 6 to 8 participants for the heterogeneous FGD.

### Data Collection

The online FGDs were held in Dutch over Microsoft Teams between September 2024 and January 2025. The duration of the FGDs ranged from 66 to 84 minutes, with a mean of 77 minutes. The homogeneous FGDs were conducted first, followed by the heterogeneous FGD. Every FGD was facilitated by two researchers: one acting as a moderator (BVD) and the other as note-taker and co-moderator (TVDB, SR, CHMVJ, and Dr Suzanne Smit). The moderator was familiar with most participants, as they were (direct) colleagues working within various PHSs.

Given the limited knowledge of IDC professionals’ views on DDW, we developed a broad and exploratory topic guide (Multimedia Appendix 1). The guide was based on two complementary theoretical frameworks: the Consolidated Framework for Implementation Research (CFIR) [10,11] and the Attitude-Social Influence-Efficacy (ASE) model [12], complemented with questions on the current status of DDW within the PHS. The CFIR was used to identify potential barriers and facilitators within the implementation process, including questions about individual and organizational barriers, facilitators, and needs. The ASE model, which focuses on individual behavioral factors, such as intention, attitude, social influence, and self-efficacy, was integrated into questions related to professionals’ knowledge, attitude, perceptions, and intentions. This version of the topic guide was tested with an epidemiologist and a nurse who did not participate later in the study. All FGDs started with an open question on the definition of data-driven IDC to collect insights into participants’ knowledge and perceptions of the definition. Afterward, the moderator provided the definition of data-driven IDC, which guided the discussions (as stated in the Introduction). The topic guide for the heterogeneous FGD aligned with that of the homogeneous groups but emphasized the current status of DDW and role distribution within the process. Across the final FGDs for each professional group, few new themes emerged, largely confirming findings from earlier discussions. In the heterogeneous FGD, no new themes were identified, suggesting that thematic saturation had been reached.

### Data Analysis

All focus group discussions were audio recorded, transcribed verbatim, deidentified, and checked for accuracy. Two researchers (BVD and TVDB) coded all transcripts independently and discussed until consensus was reached. An expert in qualitative and implementation research (JEMS) was consulted for advice when needed. The researchers began with framework coding and supplemented with thematic coding. Later in the coding process, axial coding was applied. ATLAS.ti v. 24.0 was used to facilitate the coding process and data analysis [13].

## Results

### Overview

We conducted 9 online FGDs with 36 IDC professionals, ranging from 3-6 participants (Table 1). A total of 2 homogeneous FGDs were held with medical doctors (in training), 2 with nurses and an infection preventionist, 2 with infectious disease epidemiologists, and 2 with policy advisors or officers, managers, and a project leader. The heterogeneous FGD included a medical doctor, 2 nurses, 2 infectious disease epidemiologists, and a manager. Five main themes emerged from the FGDs: (1) context of the work environment, (2) interpretation of DDW in the context of IDC, (3) added value of DDW, (4) views on team participation, and (5) perspectives on development and implementation.

**Table 1:** Characteristics of participants in 9 focus group discussions with infectious disease professionals in the Netherlands.

Characteristic			Participants (N=36)
**Profession, n (%)**			
	Infectious disease epidemiologist	10 (28)	
	Medical doctor (in training)	9 (25)	
	Nurse	8 (22)	
	Policy advisor or officer	4 (11)	
	Manager	3 (8)	
	Infection preventionist	1 (3)	
	Project leader	1 (3)	
**Sex, n (%)**			
	Female	29 (81)	
	Male	7 (19)	
**Region, n (%)**			
	South (6 PHSs^a^)	10 (28)	
	North (9 PHSs)	9 (25)	
	East (5 PHSs)	6 (17)	
	West (4 PHSs)	6 (17)	
	Central (1 PHS)	5 (14)	
Work experience at PHS, median (range)			4 years (3 months-29 years)

^a^PHS: Public Health Service.

### Context of the Work Environment Within IDC

Participants stated that DDW is currently partially implemented within IDC, with differences among PHS regions (Textbox 1). Most DDW applications are used in surveillance, workload monitoring, and quality control, such as dashboards based on multiple internal and external data sources. Discussions on data interpretation and feedback on data and results are embedded in team meetings. DDW is less applied in policy- and decision-making.

Many organizational barriers hinder the implementation of DDW. Examples include the current inadequate IDC data registration system and the lack of IT infrastructure, such as data warehouses. These factors result in insufficient IDC data collection and poor accessibility, uniformity, and quality of IDC data. Additional examples of organizational barriers include restrictive privacy policies, lack of support from management and stakeholders, insufficient manpower, lack of team consistency, and limited financial resources.

The technology is just crucial. It needs to run smoothly, be properly set up, and that’s a huge challenge. These data streams, we have so many different ones, each with its own kind of plug, and it's pretty much impossible to load them all in the same way somewhere without running into thirty different ifs and buts. So that’s really, and it has been for a long time, that’s really the challenge for data-driven work. How do you make sure you create a platform where everything comes together in a uniform way, so it can all be processed at the same time? Yeah, that’s kind of the crux of it.

Infectious disease epidemiologist

**Context of the current work environment within infectious disease control—subtheme and corresponding framework element (theme 1). Framework elements were derived from the Consolidated Framework for Implementation Research (CFIR).**

**Current status of data-driven work (DDW)**

- DDW is already partially applied in surveillance, workload monitoring, and quality control.
- DDW is less applied in policy and decision-making.
- There are regional differences in application and development.
- Public Health Services (PHSs) attempt to develop a vision on DDW in infectious disease control (IDC).

**Current cases of DDW in IDC**

- Examples of DDW include surveillance, workload monitoring, quality control, prioritization, intervention development, and testing of gut feelings and assumptions.

**Types of data currently used in DDW**

- Internal data sources: infectious disease notifications, enquiries on infectious diseases, vaccination coverage statistics, and workload data.
- External data sources: Google Trends data and population statistics.

**Current communication channels for feedback of DDW results and development**

- Feedback on ad hoc requests or investigations during meetings with the IDC team.
- Feedback to management through reports.
- Communication through newsletters to external partners.
- Announcements or information shared on a whiteboard for the IDC team.

**Barriers in the inner setting domain (organizational factors)**

- Lack of team consistency.
- Lack of a shared mission and vision on DDW within IDC.
- Inadequate systems and data infrastructure.
- Insufficient data.
- Restricted accessibility to data.
- Poor data quality.
- Lack of data uniformity.
- Conflicting interpretations of the data.

**Barriers in the outer setting domain**

- Limited financial resources.
- Lack of manpower.
- Restrictive privacy regulations.
- Challenges in collaborating with diverse stakeholders.
- Unsupportive management.

### Interpretation of DDW in the Context of IDC

Participants had different understandings of the concept of DDW (Textbox 2). For some participants, it referred to taking actions or making decisions based on data, whereas others described it as working efficiently based on real-time data. A few participants found it difficult to define DDW.

**Interpretation of data-driven work (DDW) in the context of infectious disease control—subthemes and corresponding framework elements informed by the Consolidated Framework for Implementation Research (CFIR) and the Attitude-Social Influence-Efficacy (ASE) model (theme 2).**

**Given definitions of DDW (informed by CFIR)**

- Different understandings exist regarding the concept of DDW in infectious disease control (IDC).

**Attitude toward the concept of DDW (informed by ASE)**

- DDW is not new in IDC.
- DDW is perceived as an abstract concept.
- DDW is viewed as a tool.
- DDW is seen as futureproof.
- DDW is considered to have added value in IDC.

**Attitude toward the importance of data interpretation by IDC professionals (informed by ASE)**

- Data interpretation within DDW is considered crucial.
- The interpretation of data is viewed as a team effort.
- DDW is of greatest value when quantitative data are complemented by qualitative data.

**Perception of the importance of data interpretation by IDC professionals (informed by ASE)**

- Presenting data without interpretation or context was perceived to result in misunderstanding.

**Expected disadvantages of DDW (informed by ASE)**

- DDW is sometimes viewed as the “holy grail” rather than as a tool.
- Information not captured in data is at risk of being ignored.
- Poor data quality can lead to incorrect conclusions and decisions.
- There is fear of undermining professional expertise.
- There is concern that no room is left for gut feeling in data interpretation.
- Resistance may arise from fear of job replacement.
- Strict data registration may make the job less enjoyable.
- There is fear of individual productivity tracking.
- There is a perceived risk of micromanagement.

**Experienced disadvantages of DDW (informed by ASE)**

- Data were presented and shared without sufficient context.
- Decision-making was sometimes based on data without critical reflection.
- Participants expressed conflicting feelings: trusting the data versus relying on gut feeling.
- The process was described as time-consuming due to higher administrative burden.
- Privacy regulations imposed restrictions.
- Outcomes were perceived to depend on data quality (“rubbish in, rubbish out”).
- Lack of data uniformity made comparisons challenging.

After sharing the definition of DDW as applied in this study, most participants expressed a positive attitude toward the application of DDW in IDC, as long as data are placed within its context. They mentioned that IDC professionals’ insights and gut feelings are of great importance in the interpretation of data and should not be ignored in DDW applications. Data interpretation is seen as a team-effort that requires input from nurses, medical doctors, and epidemiologists. Participants also stated that quantitative data should be complemented and interpreted with qualitative data in order to obtain accurate information. Participants see DDW as a tool that provides great opportunities in the current setting as well as in the future.

I think if you strip it down and look at it simply, then I don't think you should let just the statistics, just the data make the decisions. There really has to be that human interpretation alongside it. When we talk about data-driven healthcare, I don’t think it’s about the data making the call, the data is your foundation, and then you, as a human, look at it. And the human, like you said earlier, [Name], places it in context. A person interprets it and sees the bigger picture, because a statistical model isn’t capable of that yet. Maybe in the future, with all the developments in artificial intelligence, but for now, I think it shouldn't be the model or the statistics or the data making the decisions. It can form a solid base, sure, but right now I think humans are still better at making those calls.

Infectious disease epidemiologist

It’s not just about quantitative data, it’s also about qualitative data, of course. What’s behind those numbers? So sometimes it’s really useful to follow up. We saw that too with the vaccination data, like: hey, why are things going a bit less smoothly at that one office? Then you want to hear from them: so, how are things going at your office? What challenges are you facing? What’s causing that? You want to get that information back, in a way. So it’s always a mix of quantitative and qualitative data. And you often can’t get qualitative data from your data systems. You have to go back to the source or, if needed, do a small study to look into it.

Medical doctor

Despite the overall positive views on DDW in IDC, disadvantages were expected and already experienced. For example, the fear of undermining professionals’ expertise was expected by participants. An example of an experienced disadvantage is that data were presented without contextual explanation, which resulted in misunderstandings.

I actually had a hands-on experience with this once myself. I was working with an external party that was helping with my policy plan, and I wanted to send them a graph. I thought, this speaks for itself: the trend is clearly going down. But then my colleagues kind of gave me a hard time: like, no, you really have to include some interpretation with it, otherwise it can be misunderstood, and then you’ve got a problem. That was definitely a 'learning the hard way' moment. Even if you have the data, as a professional, you need to add that explanation, otherwise it can be misrepresented in the media or in the press.

Policy advisor

### Added Value of DDW

Participants perceived the application of DDW for several purposes in IDC, such as generating insights for surveillance, early detection of infectious diseases, and providing management information (Textbox 3). However, decision-making or initiating actions based on the insights derived from data are not yet structurally embedded. Participants explained this by citing the early development stage of DDW, lack of manpower, unclear role distribution, and the absence of a clear objective for data collection. Furthermore, participants stated that DDW could be applied more for predicting trends in infectious diseases, thereby facilitating a more proactive approach. They also suggested that DDW could be used more extensively to assess the effects of interventions on specific target populations. The availability and accessibility of data are therefore necessary conditions, which are currently lacking for several topics within IDC.

I was just thinking a bit more about that part around interpretation. You’ve got the data, and then the interpretation is really more about the substance behind it. I think we could probably play a bigger role there by taking a bit more time upfront to really consider the data and translate it more like, what does it actually mean for childcare centers, for example, or for a school, or for the general public? Or what does it mean in terms of prevention, or for vulnerable groups? That kind of translation. I get the sense that at our PHS, the focus still lies more with the data itself, and less with translating it into action or prevention.

Nurse

A positive attitude toward the results of DDW was clearly expressed by most participants. For example, they mentioned that DDW can improve quality of work and helps them work more efficiently. In addition, DDW results in making well-founded decisions. In daily practice, participants perceived progress in the development and implementation of DDW, and they mentioned that DDW becomes more enjoyable due to increasing knowledge on this topic. Several results of DDW were highlighted, such as a more structured workflow, better prioritization, and the ability to intervene proactively by early detection of infectious diseases. Participants stated that data-driven strategies add value to IDC and that the results justify the time and effort required.

The first thing that comes to mind is that at our PHS, it really provides guidance, we're doing the work in a more concrete way now. Before I started using the dashboard and looking at the numbers that way, a lot of things were handled quite ad hoc by the doctors and nurses. Like, there was a feeling or a question, and then we'd take a look at it, but not in a very structured way at a fixed moment, looking at all the notifications: what have we seen, what can we expect. So, it’s brought us more structure and also visibility into things we weren’t actively looking for. In the past it was more like: there’s an idea, we hear something, or a question comes from somewhere, and then we’d start exploring. But now, with the dashboard, you also see things you weren’t consciously searching for, but that become visible anyway.

Infectious disease epidemiologist

**Added value of data-driven work (DDW)—subthemes and corresponding framework elements informed by the Attitude-Social Influence-Efficacy (ASE) model (Theme 3).**

**Attitude toward the application of DDW**

- DDW is applicable for quality improvement, generating insights, early detection, prioritization, developing interventions, and generating management information.
- DDW is only applicable when data are available and accurate.
- DDW is not sufficiently used in decision- and policymaking.
- DDW could be more proactive rather than reactive.
- DDW could focus on the effects of interventions.
- DDW could be smarter and more efficient.
- DDW could focus more on qualitative data.

**Attitude toward the results of DDW**

- DDW results in working more efficiently.
- DDW provides complete insights into surveillance.
- DDW improves quality of work.
- DDW supports prioritization and provides management information.
- DDW enables proactive and prepared responses to unexpected situations.
- DDW results in well-founded decisions.
- DDW is worth the time investment.
- DDW can lead to greater cost-effectiveness.
- Results of DDW can be difficult to measure.

**Perception of the application of DDW**

- DDW strategies are initiated within different domains of IDC.
- DDW is mainly applied to gain insights, but these insights are not always translated into action.
- DDW is not fully applicable due to a lack of data for certain topics in IDC.

**Perception of the results of DDW**

- Progress in the development and implementation of DDW is visible.
- The time investment pays off.
- DDW has brought more structure and prioritization to IDC.
- DDW enables efficiency by generating quick insights and overviews.
- DDW allows proactive intervention through early detection of infectious diseases.
- DDW provides evidence to support decision- and policymaking.
- DDW leads to better quality of work through clear data requirements.
- Greater knowledge of DDW has made working data-driven more enjoyable.
- DDW strengthens internal collaboration.

### Views on Team Participation in DDW

According to the participants, attitudes toward and participation in DDW vary among colleagues (Textbox 4). However, in general, there is support for implementing DDW in IDC. For example, people who are positive toward DDW, interested in data, or aware of the importance of DDW are willing to commit and may even initiate data-driven strategies. They perceive it as innovative and valuable to their work. In contrast, participants mentioned that some colleagues are more skeptical and negative toward DDW, as they do not fully grasp the concept, are concerned about being too reliant on (insufficient) data, or are resistant to change. As a consequence, these colleagues were less involved in DDW as perceived by the participants. On the other hand, participants mentioned that some colleagues expressed a clear willingness to participate in DDW, yet their involvement remained limited due to being more patient-oriented, not feeling responsible for data interpretation, high workload burden, limited time, or insufficient (digital) skills.

**Views on team participation in data-driven work (DDW)—subthemes and corresponding framework elements informed by the Attitude-Social Influence-Efficacy (ASE) model and the Consolidated Framework for Implementation Research (CFIR; Theme 4).**

**Participant involvement in DDW (informed by CFIR)**

- Epidemiologists
  Are the driving force for initiating DDW in surveillance.
  Feel responsible for data analysis.
  Feel responsible for data visualization.
  Are actively participating in data interpretation.
  Are actively involved in providing feedback.
- Medical doctors
  Are actively involved in and responsible for data interpretation.
  Those interested in DDW participate actively in initiating DDW.
  Those less interested in DDW are passively involved in initiating DDW.
- Nurses
  Feel responsible for data collection and registration.
  Are actively involved in data collection and registration.
  Are less involved in data analysis and interpretation.
- Policymakers
  Are passively participating in data interpretation.
  Benefit from the results of DDW.
  Feel responsible for embedding the results, optimizing processes, and providing feedback on DDW.
- Managers
  Feel responsible for undertaking actions based on management information derived from data.
  Feel responsible for providing an adequate work environment to support DDW.

**Participants’ skills for DDW (informed by CFIR)**

- Digital skills vary among IDC professionals.
- Epidemiologists have expertise in data management and analysis.
- Interpreting data is not the core task of nurses or policymakers.
- Managers interpret data differently from IDC professionals.
- There is a lack of IT expertise within IDC teams.

**Attitude toward the importance of overall team participation (informed by ASE)**

- There is no consensus on the necessity of overall team participation.
- Each IDC profession has a different role and responsibility within DDW.
- The entire IDC team must be informed about DDW developments.

**Team perspective on DDW (informed by ASE)**

- Positive
  DDW is appreciated for its innovation and opportunities.
  There is a strong will to apply DDW within IDC.
  DDW is valued when processes are explained and outcomes are fed back.
- Negative
  DDW remains an unknown concept for some IDC professionals.
  Resistance arises due to changes in work processes.
  Skepticism toward data-driven decision-making exists due to mistrust in the data.
  The purpose or usefulness of DDW is sometimes unclear.
- High and unrealistic expectations exist regarding the application of DDW.

**Team participation in DDW (informed by ASE)**

- There is overall strong commitment among most IDC professionals.
- People are involved when:
  They are naturally interested in data.
  Someone within the team acts as a driving force.
  They see the added value of DDW.
  Updates are given and awareness is raised.
  Tools such as dashboards are accessible.
- People are less involved when:
  They are patient-oriented rather than data-oriented.
  Participation requires additional effort and time.
  Workload is high and priorities lie elsewhere.
  They do not feel responsible for data interpretation and rely on others.
  They are conservative and find their existing work approach sufficient.

**Barriers in the individual domain (informed by CFIR)**

- Lack of time to invest in DDW.
- The burden of data registration.
- Lack of knowledge about DDW.
- Negative sentiment toward DDW.

I notice there’s a bit of a divide. Part of the team is interested and open to it. They might still find it a bit complicated, but they’re motivated to work with it. Still, even for them, it takes real effort to actually weave it into their day-to-day work. Then there are also colleagues, you can tell, who are more focused on direct contact with patients or on phone calls and helping people. And for them, things like surveillance or entering data sometimes feel more like a side task, so to speak. Even though they do realize it’s important input, it’s just not really where their energy lies. So yeah, it varies.

Medical doctor

Participants’ views differed on the necessity of overall team participation for the implementation of DDW to succeed. However, they agreed that all team members should be informed about developments and could contribute based on their profession, knowledge, and skills.

Multiple steps of DDW were identified: initiation of DDW strategies, data collection, analysis and visualization, interpretation, action, and feedback. Epidemiologists are seen as the key drivers of DDW strategies and are actively involved in data analysis, visualization, interpretation, and feedback. Medical doctors lead data interpretation, but their involvement in initiating DDW depends on personal interest, time, and prioritization. Nurses focus on data collection and registration but are less involved in analysis and interpretation; however, an active role of nurses in interpreting data was seen as an added value. Policymakers are less involved in all process steps but benefit from DDW outcomes and see themselves as responsible for embedding results, optimizing processes, and ensuring feedback. Managers are responsible for taking action based on management information derived from data and for supporting DDW by facilitating a suitable work environment. Participants expressed that, ideally, managers do not take part in interpreting data, as it is not part of their expertise.

In our case, we’ve divided things into different sub-teams. A signal might come in first to the medical staff or nurses. That signal grows, and then another sub-team, the one that focuses more on data, gets involved because they pick up on it. Once they gather more information, there might be collaboration with yet another sub-team that works more on policy and prevention. So you can really see how a signal that first comes in through, say, a nurse, eventually moves through the whole team and results in a preventive measure or a policy-level intervention. It’s an ongoing process and there’s always room for improvement, but I do see a really positive development in how this works within our team.

Manager

### Perspectives on Development and Implementation of DDW Within IDC

Currently, the implementation of DDW within IDC occurs primarily at the organizational level (Textbox 5). However, participants expressed that it should be approached nationally and supported by a shared vision on DDW created and defined by IDC professionals. The importance of openly and widely sharing DDW results and goals to ensure collaboration with other (external) stakeholders was emphasized, as participants considered this essential for the successful adoption of DDW. Additionally, while participants frequently stressed the importance of evaluating DDW, they noted that it was not yet structurally embedded. Although most participants were positive toward DDW, they stressed that implementation should not come at the cost of daily routine work, such as case and outbreak management, which medical doctors and nurses considered their primary responsibility.

I think we really need to develop a much clearer vision on this like, where do we ultimately want to go, what’s our goal? Right now, each PHS is kind of setting its own dot on the horizon. Or some don’t even have one, they’re just doing their thing and we’ll see where it ends up. But I think as a professional group, as infectious disease control teams in the Netherlands, we really need to have a shared vision: where do we want to go?

Medical doctor

Several facilitators were identified by the participants. On an individual level, adoption of a data-driven approach was supported by intrinsic motivation, adequate knowledge, and sufficient time. Furthermore, the team and management also played important roles in implementation. When the team and management are aware of and supported data-driven strategies and ensured continuity of manpower, adoption and implementation benefited. On a process level, implementation ran more smoothly when data agreements were established, a shared mission and vision on DDW were in place, collaboration with other stakeholders was ongoing, and feedback loops on results and processes were embedded.

It’s really about how you bring about a cultural shift. That never happens in a day. I think you need to do it in a low-key way, not just push it top-down onto a team all at once. You probably need a few early adopters who are really enthusiastic and embrace it, who drop it into the team now and then so that others start to see: hey, this is actually useful. When people start saying, ‘Now that we’ve seen this, we’re also noticing an increase in reports,’ then it starts to click. Honestly, I think what we’re doing now in our team is a great example, sharing a monthly overview. That way, even those who aren’t that into data can still see: this is going up, this is going down. It becomes simple and intuitive. Because often with mandatory IT changes, people just see it as an extra task or something complicated. And that’s exactly what it shouldn’t be. It should be something that makes the work easier, more efficient, and not time-consuming. I think when that’s the case, professionals really are open to it.

Policy advisor

To further develop DDW within IDC, participants voiced several needs for the future. Primarily, a robust and accurate system for registration, surveillance, preparedness, and management is needed. This system should provide real-time insights based on internal and external data sources at local, regional, and national levels, facilitating an ongoing feedback loop on DDW results. In addition, this system could enable data to be used more for action rather than for reflection only.

Participants emphasized the need for well-structured, multidisciplinary teams to successfully adopt DDW. Teams should have sufficient manpower, access to education, and a shared willingness to work in a data-driven manner. In addition, a clear distribution of roles and responsibilities within the team was viewed as crucial by the participants—for example, appointing someone to initiate and oversee data-driven strategies, as well as ensuring dedicated involvement of data science and IT professionals.

A strong need expressed by the participants was national-level coordination that defines a vision and a mission for DDW, preferably formally endorsed and financially supported by the directors of PHS. According to participants, such coordination would enable the development of clear work protocols for data registration and guidance on privacy regulations, thereby facilitating the required collaboration among the PHSs.

When asked what we need for the future to work in a data-driven way, I’d actually like to zoom out first. You need a supportive political climate that’s willing to invest in this for the long term — not just for two or three years. That way, you have the resources you need. And with those resources, you can hire skilled IT professionals and epidemiologists at every PHS, so that dashboards can be developed everywhere.

Policy advisor

**Perspectives on the development and implementation of data-driven work (DDW) within infectious disease control (IDC)—subthemes and corresponding framework elements informed by the Attitude-Social Influence-Efficacy (ASE) model and the Consolidated Framework for Implementation Research (CFIR; Theme 5).**

**Attitude toward the implementation of DDW (informed by ASE)**

- Implementation of DDW needs to occur at both the organizational and national levels.
- The implementation process of DDW should be established beforehand.
- Collaboration with external stakeholders is essential for effective DDW.
- Results and goals of DDW should be broadly and transparently shared.
- A shared vision of DDW among IDC professionals should be developed.
- Evaluation of DDW strategies is important.
- Implementing DDW must not come at the expense of daily routine work.

**Facilitators in the individual domain (informed by CFIR)**

- Intrinsic motivation to adopt DDW.
- Availability of time to invest in DDW.
- Education and training on DDW.

**Facilitators in the inner setting domain (organizational factors; informed by CFIR)**

- Awareness of DDW within the IDC team.
- Continuity within the IDC team.
- Support for DDW from the IDC team.
- Agreements on data registration practices.
- A shared mission and vision on DDW within IDC.
- A feedback loop on DDW results and processes.

**Facilitators in the outer setting domain (informed by CFIR)**

- Support from management.
- Collaboration on DDW with external stakeholders.
- Sufficient manpower to sustain DDW initiatives.

**Participants’ needs to improve the implementation and adoption of DDW (informed by CFIR)**

- An accurate system for registration, surveillance, preparedness, and management information that:
  Provides a comprehensive overview.
  Generates insights.
  Combines internal and external data sources.
  Operates in real time.
  Is available at the Public Health Service (PHS), regional, and national levels.
- Data that support action, not only reflection.
- Sufficient manpower for sustainable DDW efforts.
- Dedicated involvement of data science and IT professionals.
- An assigned professional responsible for initiating and overseeing DDW within IDC.
- Clear role and responsibility distribution among IDC professionals.
- A defined mission and shared vision for DDW within IDC.
- Willingness to change current ways of working.
- Clear work protocols for data registration.
- Clear guidance on privacy regulations.
- Coordination at the national level.
- Increased collaboration between PHSs.
- Consensus on DDW strategies among PHS directors.
- Long-term financial resources for DDW implementation.
- Education on DDW for IDC professionals.
- An ongoing feedback loop on DDW results.

## Discussion

### Principal Findings

We aimed to explore IDC professionals’ knowledge, attitudes, and perceptions toward DDW in IDC, and to assess their needs, facilitators, and barriers for successful implementation of this approach in daily practice. Five main themes were identified: (1) context of the work environment, (2) interpretation of DDW in the context of IDC, (3) added value of DDW, (4) views on team participation in DDW, and (5) perspectives on development and implementation of DDW. Within the first theme, we found that DDW is currently partially implemented within IDC and is applied for various purposes, with differences among PHS regions. Data are primarily used for reflection on surveillance, workload monitoring, and quality control, whereas its application in decision-making and initiating actions remains limited. Several barriers to the adoption and implementation of DDW were mentioned. Regarding the second theme, participants described different definitions of DDW, illustrating the complexity of the concept. Within the third theme, participants expressed a positive attitude toward DDW and its results, as it provided substantial value to the field of IDC. Fourth, we found that overall, participation in DDW among colleagues within IDC varied. Engagement in DDW depended on role, interest, workload, time, knowledge, and willingness to change. Within the last theme, we found that although many facilitators for DDW implementation were mentioned, participants saw room for improvement. They recommended a national approach guided by a shared vision, defined professional’s role assignments, and clear protocols on data registration and privacy regulations.

### Comparison to Previous Work

In this study, participants provided varying definitions of DDW, and some struggled to define the concept. This suggests that a clear and shared understanding of DDW is lacking within IDC, which may partly explain why data are not yet systematically used for decision-making and for initiating actions in IDC. In addition, we found that the absence of a clear objective for data collection further contributes to the current challenge of using data to drive action. These findings highlight the need for a clear and common understanding of the process steps involved in DDW to ensure that data can be effectively used for decision-making and initiating actions.

Chiolero and Buckeridge [14] described a model outlining the steps in data processing for public health surveillance. This process starts with data collection, followed by data transformation and analysis, information production, dissemination, and finally, decision-making. In another study [15], the DIEK pyramid was mentioned, which outlines the continuum from data to information, to evidence, and then to actionable knowledge. Dammann [15] emphasized that evidence must be relevant and useful in order to be translated into knowledge that justifies action. These models clarify the process steps in DDW, aiming to collect data that can be translated into relevant and actionable knowledge, which can contribute to a clear and shared understanding of DDW. This shared understanding forms the foundation for developing a shared vision for the further implementation of DDW, including clear role assignments to cover all processes related to DDW.

While not all IDC professionals are willing to participate in DDW, and views differed on whether full team participation is necessary, it was perceived as helpful when colleagues were enthusiastic about DDW. This study showed that every IDC professional has a role within the different steps of DDW: initiation of DDW strategies, data collection, analysis and visualization, interpretation, action, and feedback. Although most participants acknowledged their own role and responsibility, they noted that the absence of a clear role distribution hinders colleagues’ participation and results in conflicting expectations of responsibilities during the DDW process. It is known that the lack of clear roles and responsibilities is a barrier to decision-making, especially in public health emergencies [16]. Consequently, a defined distribution of roles and responsibilities was a clear need identified by participants. It is recommended to specify and communicate all roles and responsibilities in the DDW process to IDC professionals, as DDW is a joint team effort.

Another main theme in this study was participants’ perspectives on DDW development and implementation, highlighting barriers, facilitators, and needs. One of the barriers, and therefore needs, was the lack of a data registration system and IT infrastructure. This is not only a barrier for DDW in IDC but has also been identified as a barrier in infectious disease surveillance in general [6]. Inaccessible and insufficient data, due to a lack of consensus on data registration, complicates data analysis and interpretation and therefore result in difficulties in DDW. Having a robust and reliable data registration system is one of the key elements for successful implementation of DDW [5,17-20]. The importance of integrating multiple data sources, such as regional and national data sources, with local internal data has been emphasized [18,20]. However, this study showed that the lack of clarity in privacy regulations hinders this development, which has been described in other studies as well [2,8]. It is essential for future implementation of DDW that the data registration system enables IDC professionals to work uniformly and provides insights at local, regional, and national levels.

Participants experienced many other known barriers for implementing data-driven decision-making, such as insufficient (financial) resources, lack of knowledge, high staff turnover, limited time, and resistance to change [17,19]. However, these factors also acted as facilitators when addressed positively. Participants expressed that a shared willingness, clear vision, and national coordination on DDW would benefit adoption and implementation. According to the literature, supportive management is a key facilitator of data-driven decision-making, as management is more willing to invest time and money [17,20]. Ultimately, this enables IDC professionals to initiate and develop data-driven strategies by investing time and knowledge, thereby creating the needed shared willingness.

A key strength of this study lies in the approach of conducting FGDs segregated by profession, followed by a mixed-profession FGD. This method allowed participants to share and explore their views on DDW in IDC with colleagues from the same profession, providing in-depth insights into the perspectives of different professional groups. Conducting the final FGD with mixed professions contributed to a rich discussion in a setting that reflected the multidisciplinary nature of the field of IDC. Another strength of this study is the extensive duration of the interviews, which enabled us to broadly explore perspectives on DDW in IDC and gain a deep understanding of participant’s attitudes, perceptions, and needs. Furthermore, the topic guide was based on the ASE model, which focuses on individual behavioral components, as well as the CFIR framework, which explores the implementation process. This empowered us to apply a structured and theory-driven approach throughout the study. Given the limited previous research on this topic, our approach enabled an extensive exploration on of IDC professionals’ perspectives on DDW, thereby addressing a critical gap in the literature.

### Limitations

Some limitations of this study have to be taken into account. First, selection bias may have occurred in the recruitment of participants. Since most participants had affinity for DDW, the viewpoints of IDC professionals who do not share this interest may be lacking in our findings. However, the input of the participants included critical notes and many barriers, providing a well-rounded perspective on DDW within IDC. In addition, many of the participants, especially the epidemiologists, have relatively limited work experience at PHSs, as most of them joined the PHS when the Ministry of Health, Welfare and Sport introduced financial support for PHSs in 2023. As a consequence, the perspectives of epidemiologists with more extensive work experience are less represented in our results. On the other hand, the geographical and organizational spread of our participants enabled us to capture a thorough overview of DDW in IDC. Another limitation is that the recruitment of participants from certain professions was challenging and last-minute cancelations occurred, which resulted in some FGDs comprising only 3 participants. However, we conducted multiple FGDs with the same professions to ensure validation of our results.

### Conclusions

In this study, we found that data-driven IDC is generally viewed positively by IDC professionals. Nonetheless, there remains substantial room for improvement regarding its adoption and implementation, especially in translating data into action. Factors such as the current work environment, a lack of a clear definition and vision of DDW, complex attitudes toward the concept and results of DDW, social dynamics within IDC teams, and other barriers hinder this process. For future implementation, we recommend establishing a clear shared vision for DDW within IDC. This vision should include an operational definition of DDW that outlines the entire process and clearly specifies the roles and responsibilities within each step to ensure that data translate into action. Empowering IDC professionals to work in a data-driven manner is essential for strengthening proactive responses and improving preparedness for future public health challenges, including pandemics.

## Appendix

Multimedia Appendix 1 Topic guide for focus group discussion on data-driven infectious disease control.

Multimedia Appendix 2 ChatGPT conversations.

## Abbreviations

ASE: Attitude-Social Influence-Efficacy
CFIR: Consolidated Framework for Implementation Research
DDW: data-driven work
FGD: focus group discussion
IDC: infectious disease control
PHS: public health services
WMO: Wet medisch-wetenschappelijk onderzoek met mensen

## Data Availability

The transcripts generated and analyzed during this study are not publicly available due to confidentiality reasons, but deidentified extracts are available from the corresponding author on reasonable request.
