# Supplementary material for: Data-Driven Infectious Disease Control: Qualitative Study of Professionals’ Attitudes, Barriers, and Needs
Source: J Med Internet Res. 2025 Nov 17;27:e81036. doi: 10.2196/81036 (PMC12670049; doi:10.2196/81036)
Supplement: Multimedia Appendix 1 [file jmir_v27i1e81036_app1.pdf]

# Multimedia Appendix 1 - Topic guide focus group discussion

**Knowledge**

**Understanding data-driven working**

- What does data-driven working mean to you?
  - What does it look like in your daily work?
- What falls under data-driven working?
  - Can you give some examples?

*Explain what we mean by data-driven working in the context of this study to ensure all participants are talking about the same thing.*

**Current Practice**

- Is data-driven working currently applied within your PHS?
  - If yes, can you give some examples?
    - What does it look like in daily work?
    - Does everyone participate?
  - If no, what is the reason for that?
- How are decisions regarding policy or decision-making currently made?
  - Describe the process: who is involved, which elements are crucial or less important?

**Attitude**

**Personal attitude**

- What is your opinion of data-driven working?
  - Do you engage in data-driven working yourself?
- Does data-driven working add value within infectious disease control?
  - Does it add value for you as a professional in your daily work?
  - Is it valuable for all disciplines?

**Advantages and disadvantages**

- What are the advantages of data-driven working?
- What are the disadvantages of data-driven working?

**Role distribution**

- How do you see your own role within data-driven working?
    - Do you take on this role yourself?
    - What do you need to be able to do that?
    - Which part of the chain are you involved in? From signal/notification → policy making
- How does the field, in general, view data-driven working within infectious disease control?
    - Is it important that everyone on a team participates in data-driven working?
- Do you see differences between professionals in the extent to which they apply data-driven working in their daily work?
    - Does everyone have the same role, or does it differ by profession?

**Intention**

- What do you think is needed to embrace a more data-driven approach?
    - Do you feel a need for a vision regarding data-driven working ?
- What would your ideal data-driven working environment look like?
    - What would be needed to achieve this?
    - Do you have everything available to work in a data-driven way?

**Closing and Feedback**

- What are the most important points you noticed during this discussion?
- Are there topics we haven't covered that you think are important?
- How can the outcomes of this focus group contribute to improving data-driven working and information provision in your work?
