# Supplementary material for: Data-Driven Infectious Disease Control: Qualitative Study of Professionals’ Attitudes, Barriers, and Needs
Source: J Med Internet Res. 2025 Nov 17;27:e81036. doi: 10.2196/81036 (PMC12670049; doi:10.2196/81036)
Supplement: Multimedia Appendix 2 [file jmir_v27i1e81036_app2.pdf]

Multimedia Appendix 2 – ChatGPT conversations

https://chatgpt.com/share/68dfd652-519c-8005-b6ef-7f196e8b1210

https://chatgpt.com/share/68dfd3a3-de00-8005-a81c-30f861611032

https://chatgpt.com/share/68dfd60b-72cc-8005-af63-326f10f192d6

https://chatgpt.com/share/68dfd4db-d0dc-8005-bf45-6e6a8c6884c9
